# The lipogenic enzyme acetoacetyl-CoA synthetase and ketone body utilization for denovo lipid synthesis, a review

**DOI:** 10.1016/j.jlr.2023.100407

**Published:** 2023-06-23

**Authors:** James D. Bergstrom

**Affiliations:** Mountain Stream Communications, Hillsborough, NJ, USA

**Keywords:** acetoacetate, denovo lipid synthesis, ATP citrate lyase, lipid interconversion, glucose sparing, anabolic role for ketone bodies, cholesterol synthesis

## Abstract

Acetoacetyl-CoA synthetase (AACS) is the key enzyme in the anabolic utilization of ketone bodies (KBs) for denovo lipid synthesis, a process that bypasses citrate and ATP citrate lyase. This review shows that AACS is a highly regulated, cytosolic, and lipogenic enzyme and that many tissues can readily use KBs for denovo lipid synthesis. AACS has a low micromolar *K*_*m*_ for acetoacetate, and supply of acetoacetate should not limit its activity in the fed state. In many tissues, AACS appears to be regulated in conjunction with the need for cholesterol, but in adipose tissue, it seems tied to fatty acid synthesis. KBs are readily utilized as substrates for lipid synthesis in lipogenic tissues, including liver, adipose tissue, lactating mammary gland, skin, intestinal mucosa, adrenals, and developing brain. In numerous studied cases, KBs served several-fold better than glucose as substrates for lipid synthesis, and when present, KBs suppressed the utilization of glucose for lipid synthesis. Here, it is hypothesized that a physiological role for the utilization of KBs for lipid synthesis is a metabolic process of lipid interconversion. Fatty acids are converted to KBs in liver, and then, the KBs are utilized to synthesize cholesterol and other long-chain fatty acids in liver and nonhepatic tissues. The conversion of fatty acids to cholesterol via the KBs may be a particularly important example of lipid interconversion. Utilizing KBs for lipid synthesis is glucose sparing and probably is important with low carbohydrate diets. Metabolic situations and tissues where this pathway may be important are discussed.

Acetoacetyl-CoA (AcAc-CoA) synthetase (AACS) (AcAc-CoA ligase, Enzyme Commission no.: 6.2.1.16) is a cytosolic lipogenic enzyme found in many tissues involved in lipid synthesis (1, 2, 3, 4, 5, 6, 7, 8, 9, 10, 11). It catalyzes the coupling of acetoacetate (AcAc), one of the ketone bodies (KBs), to CoA in the reaction shown below to produce cytosolic AcAc-CoA.

AcAc+CoA+ATP→AcAc−CoA+AMP+pyrophosphate

The cytosolic AcAc-CoA can be utilized directly for the synthesis of cytosolic HMG-CoA and then for cholesterol synthesis, or it can be cleaved by cytosolic AcAc-CoA thiolase to produce cytosolic acetyl-CoA (Ac-CoA), which can then be used for denovo fatty acid synthesis, the elongation of fatty acids, and the synthesis of other lipids (Fig. 1). In cells and tissues where AACS is present, denovo lipid synthesis cannot only start with citrate and ATP citrate lyase (ACLY) but can also start from AcAc and AACS. AACS is the key enzyme in the process where KBs can serve as substrates for denovo lipid synthesis. Denovo synthesis of lipids via this enzyme and pathway bypasses citrate and ACLY. Understanding this pathway for KB utilization for lipid synthesis and metabolic situations and where and when this pathway may contribute is important to the understanding of lipid synthesis.

**Fig. 1:**
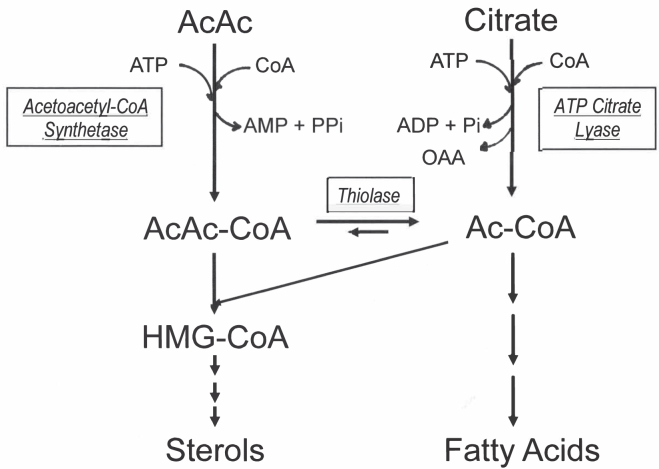
Denovo lipid synthesis from acetoacetate and citrate. Both AcAc and citrate can be used in the cytosol as substrates to provide AcAc-CoA and Ac-CoA that can be used in the cytosol for denovo lipid synthesis. AcAc enters the system via acetoacetyl-CoA synthetase producing AcAc-CoA. Thiolase can then convert this to two Ac-CoAs. Citrate enters the system via ATP citrate lyase to produce Ac-CoA.

AACS has been shown to be a highly regulated enzyme found in developing brain during myelination (6), adult liver (2, 3, 4, 5, 12, 13), adipose tissue (2, 9, 10, 12), lactating mammary gland (7, 14), tumor cells (15), and other lipogenic tissues (4, 16). It has been purified (5), its sequence derived (17), promoter region studied (18, 19), expressed (8), and shown to be essential for neural development (10). The utilization of KBs for lipid synthesis has also been studied in many of these same tissues, and the KBs have been shown to be readily utilized for denovo lipid synthesis in them. But the work in this area has been troubled by a lack of understanding of what is the physiological role of using KBs as substrates for lipid synthesis (20).

Research and publications on AACS and KB utilization for lipid synthesis has been ongoing since the early-1950s (21); however, the work on AACS and the utilization of KBs for lipid synthesis in general have never been fully reviewed (to the knowledge of the author). Numerous articles discussing denovo lipid synthesis do not mention AACS and this pathway and the possibility of bypassing citrate and ACLY in denovo lipid synthesis (22, 23). Understanding and considering this process and pathway may be of particular importance now because of the strong interest in developing drugs that regulate denovo lipid synthesis including the development of ACLY inhibitors (22, 23). One ACLY inhibitor, bempedoic acid, has been approved as a cholesterol-lowering agent, and there are efforts to produce other ACLY inhibitors for the treatment of a variety of lipid-related diseases and as anticancer agents (22, 23, 24, 25, 26). This raises the question of whether these agents that target ACLY will work in metabolic situations where denovo lipid synthesis via the AACS is a major ongoing process and the role of ACLY in denovo lipid synthesis is minimalized?

This article will first review AACS and KB utilization for lipid synthesis, the metabolic pathways for this process, and their regulation. Then the role this may have in denovo cholesterol and fatty acid synthesis, the tissues, and metabolic situations where it is or may be of importance will be discussed. An important point recently made by Aguilo *et al.* (8) is that AACS is a high-affinity enzyme with a low micromolar *K*_*m*_ for AcAc, and there is plenty of AcAc available to supply the process of KB utilization for denovo lipid synthesis in virtually any metabolic state including the fed animal and not just in states when KBs are elevated such as in diabetes or fasting. Later, it will be hypothesized that a major physiological role of this pathway is the interconversion of lipids and that KBs need to be thought of as anabolic substrates as well as catabolic ones. This anabolic role of the KBs is separate from and not necessarily connected to their catabolic role in energy production. Metabolic and nutritional states where the anabolic role of KBs could be of importance will be discussed as well as tissues where this process may be important. Last, how this pathway and the process of KB utilization for denovo lipid synthesis may impact the utility of agents that target ACLY will be discussed.

## Studies on AACS

AACS is a cytosolic enzyme involved in lipid synthesis. It is found in greatest abundance in lipogenic tissues, such as adult liver (1, 2, 3, 4, 5, 6, 8, 12), developing brain (2, 6), lactating mammary gland (7, 14), and adipose tissue (9, 10, 11, 13). Its activity was first measured by Stern in 1971 (1) in rat liver. Its developmental pattern in brain (6) and liver (2, 3, 27) correlates with the development of lipogenesis in these tissues. For instance, it is high in developing brain during the period of myelination and then is much lower in adult brain when less lipid synthesis is occurring (2). In rat liver, AACS activity is low in infancy until weaning and then increases to adult levels (2, 3, 27). In mammary gland of the female rat, the activity follows that of lipid synthesis that is needed to produce milk (2). The enzyme activity is absent from mammary gland in the virgin adult female, at low levels (1–2 μmol C2 units/h/g tissue) in the pregnant female just before birth and increase 5- to 10-fold at birth and remains high through the nursing period and then drops to very low levels about 5 days after weaning (2). AACS activities and AACS immunoreactive proteins are low in tissues not noted for high levels of denovo lipogenesis, such as adult lung, spleen, heart, and kidney (5).

The enzyme was purified from rat liver, and the complementary DNA clone encoding the enzyme was isolated (17, 28). It has an open reading frame of 2,019 nucleotides that encode an amino acid sequence of 672 amino acid residues with a molecular weight of 75,039 Da (17). The purified protein had a molecular weight of 71,000 Da. The presence of four in-frame ATG codons from nucleotides 109–195 raiseS the possibility of the alternative translation or post-translational modifications (17, 28).

### Regulation of AACS

AACS is a high-affinity enzyme with a low *K*_*m*_ for AcAc. Ito *et al.* (28) measured the *K*_m_ of the purified rat enzyme to be 8 μM, the *K*_*m*_ for AcAc for the expressed and purified human AACS was measured to be 37.6 μM by Aguilo *et al.* (8), and Bergstrom *et al.* (3) estimated the *K*_*m*_ to be 54 μM in rat liver cytosol. These *K*_*m*_ values are low as compared with the plasma concentration of AcAc (minimum around 100 μM) in almost any metabolic situation including the fed state. Aquilo *et al.* (8) concluded that AACS, a high-affinity enzyme, is active in the liver in the fed state when the liver is not producing large amounts of KBs, and it can utilize the low levels of the KBs that are characteristic of a fed animal. Since AACS is present in many lipogenic tissues and adequate levels of KBs are present in most circumstances, not just in ketotic states, the flux of AcAc being utilized for lipid synthesis via AACS must be controlled by other means than just the supply of AcAc.

The *K*_*m*_ for CoASH was measured to be 10 μM (28) for the enzyme purified from rat liver and 2.3 μM for the recombinant human AACS (8). Substrate inhibition with CoASH was seen in both studies above 50 μM CoASH for the rat liver enzyme and above 15 μM for the recombinant human enzyme. In some studies, the level of activity of AACS in tissues may have been underestimated because the AACS assays that were used had concentrations of CoASH (250 μM) that were inhibitory (29). l-(+)-3-hydroxybutyrate can also serve as a substrate at about 20–50% of the rate of AcAc (12, 28).

There can be large changes in AACS activity that would affect the flux through the pathway. In rat liver, AACS activity decreases with fasting (3, 5, 6). The rat liver enzyme exhibits a 10-fold diurnal variation in activity (3) that is related to a meal feeding behavior, and it has been demonstrating that meal feeding enhances AACS activity in liver (4, 8, 29). AACS activity is reported to be 3-fold higher in liver of adult female rats compared with adult male rats (27).

Hepatic AACS activity is highly regulated by modulators of cholesterol synthesis (Table 1) (4). AACS activity was suppressed 85% in rats fed a diet containing 7.5% cholesterol compared with controls. Feeding cholestyramine-induced activity 2.8-fold above untreated controls, including lovastatin, an HMG-CoA reductase inhibitor, in the diet induced activity 3.8-fold, and cholestyramine plus lovastatin induced activity 6.6-fold to 30.8 μmol/h/g. There was a 44-fold difference in activity between cholesterol-fed animals and animals fed cholestyramine plus lovastatin (4). Administering mevalonic acid lactone to rats also decreased AACS activity (4). These changes in AACS activity closely correlate with changes in the activities of HMG-CoA synthase and HMG-CoA reductase but not AcAc-CoA thiolase (Table 1) (4). Similar results were later reported by Ito *et al.* (5). AACS activity in isolated rat hepatocytes was suppressed by 25-hydroxycholesterol in parallel with HMG-CoA reductase (30). HMG-CoA reductase like AACS also exhibits a 10-fold diurnal variation in activity in rats with its peak activity at the midpoint of the dark cycle (3, 31). Salam *et al.* (32) looked at the stimulation of hepatic cholesterol biosynthesis by oleate in perfused rat liver. Perfusion with oleate stimulates hepatic VLDL production and the biosynthesis of lipids needed to form the VLDL particles. Oleate will readily be incorporated into triglycerides (TGs) stimulating VLDL production, but denovo cholesterol synthesis will also be needed to supply the cholesterol needed to form the VLDL particles. Oleate perfusion of livers from fed animals compared with perfusions without oleate increased AACS activity 2.2-fold while also increasing cytosolic HMG-CoA synthase 1.4-fold, cytosolic AcAc-CoA thiolase 2.2-fold, and HMG-CoA reductase 2.6-fold. In all these studies, AACS activity changed in parallel with HMG-CoA reductase and cytosolic HMG-CoA synthetase activities (4, 30, 32). In liver, AACS activity appears to be closely tied to cholesterol synthesis. Salam *et al.* (33) in a second study found that oral administration of olive oil increased hepatic AACS activity 3-fold, cytosolic HMG-CoA synthase 1.5-fold, cytosolic AcAc-CoA thiolase 2-fold, while not affecting the activity of HMG-CoA reductase. They concluded that “the enzymes that supply the HMG-CoA required for hepatic cholesterogenesis are regulated in parallel by a physiological substrate, fatty acid, independent of HMG-CoA reductase under these conditions” (33). These data suggest that dietary fat may regulate AACS and the utilization of KBs as substrates for cholesterogensis.

**Table 1:** The effect of feeding cholesterol, cholestyramine, lovastatin, and cholestyramine plus lovastatin on rat hepatic AACS, HMG-CoA synthase, HMG-CoA reductase, and AcAc-CoA thiolase

	AACS	Cytosolic HMG-CoA synthase	HMG-CoA reductase	Cytosolic AcAc-CoA thiolase
Additions to diet	µmol/h/g			

Fed controls	4.7 ± 0.4	5.2 ± 0.5	3.3 ± 0.3	11.9 ± 3.0
7.5% Cholesterol fed	0.7 ± 0.1	1.3 ± 0.1	0.39 ± 0.12	5.9 ± 0.7
5% Cholestyramine	13.3 ± 0.8	12.4 ± 0.6	21.0 ± 2.1	17.7 ± 2.0
0.1% Lovastatin	17.8 ± 3.8	17.3 ± 2.4	17.4	ND
Cholestyramine plus lovastatin	30.8 ± 2.7	31.5 ± 1.9	174 ± 6	35.2 ± 2.3

Reproduced with permission from Bergstrom *et al.* (4).

ND, not determined.

Modulation of AACS activity by cholesterol homeostasis may occur in other tissues besides liver. The precursor pool for the synthesis of steroid hormones by adrenals is normally supplied by cholesterol derived from plasma lipoproteins (34). When rats are treated with 4-aminopyrazolopyrimide, a treatment that decreases levels of plasma lipoproteins and deprives adrenals of their supply of cholesterol, denovo cholesterol synthesis in the adrenals is increased (34). With 4-aminopyrazolopyrimide treatment, rat adrenal AACS activity increased 7.3-fold and HMG-CoA reductase activity increased 15-fold demonstrating regulation of AACS activity in adrenals by its need for cholesterol (4).

AACS mRNA levels were shown to be regulated by modulators of cholesterol synthesis in mouse hepatocytes (18). The promoter region of *AACS* was found to interact with SREBP-2, and SREBP-2 is a key transcriptional factor for the regulation of AACS gene expression (18). An AACS knockdown in mice, which utilized a short hairpin RNA that targeted mouse *Aacs*, significantly reduced AACS expression and resulted in a decrease in total serum cholesterol of 28%. The AACS knockdown had increased mRNA levels of HMG-CoA reductase (18). These results add support to the evidence that hepatic AACS is an important enzyme involved in cholesterol homeostasis (4, 18).

AACS activity in rat adipose tissue was first reported to be at a low level that was reduced by starvation by Buckley and Williamson (2). In contrast, Ito *et al.* (5) found the specific activity in the cytosol of adipose tissue from adult female rats to be the higher than any other tissue they measured including liver and brain. Yamasaki *et al.* (35) reported that AACS mRNA levels were particularly abundant in subcutaneous white adipose tissue of male rats after weaning and were at much higher levels than subcutaneous white adipose tissue from females in white adipose tissue. AACS and KB utilization for lipid synthesis appears to be tied to adipocyte differentiation and the synthesis of fatty acids rather than to the synthesis of cholesterol (9, 18, 19, 35). AACS mRNA was low in 3T3-L1 cells prior to differentiation. Upon exposure to differentiation inducers, AACS mRNA increased. HMG-CoA reductase mRNA was hardly detected before and after differentiation, while the expression pattern for Ac-CoA carboxylase-1 mRNA increased and peaked together with AACS mRNA (35). The promoter region of the *AACS* gene was found to contain a C/EBP (CCAAT/enhancer binding protein) binding site, and its expression was controlled by C/EBPa during adipogenesis (9). C/EBPa is a regulator of adipogenesis and lipogenesis during adipocyte differentiation (9). The promoter region of the *AACS* gene has also been shown to contain a binding site for peroxisomal proliferator-activated receptor-gamma, a known master of adipogenesis (19). *AACS* knockdown experiments in 3T3-Li cells significantly reduced the rate of adipocyte differentiation suggesting that AACS is an important factor in 3T3-L1 differentiation and adipocyte lipogenesis (18).

When AACS was purified from rat liver, anti-AACS antibodies were prepared and used to measure the amount of immunoreactive protein in various preparations from rat tissues. This was compared with the measured activity of AACS in those same preparations (5). It was found that the ratio of AACS activity/milligram immunoreactive protein varied over 6-fold with the lowest value in kidney and the highest from the liver of animals fed cholestyramine (5). This suggests that AACS could exist in different forms that are more or less active and that its activity may be regulated by other mechanisms than just the levels of AACS protein. Other enzymes of lipogenesis such as HMG-CoA reductase (36) and Ac-CoA carboxylase (37) can be regulated by phosphorylation/dephosphorylation. The potential regulation of AACS by activation/inactivation or by different forms of AACS may be an area that warrants future research.

Another level of regulation for AACS that may be of metabolic importance is the finding that fatty acyl-CoAs are noncompetitive inhibitors of rat liver AACS (38). Palmitoyl-CoA (*K*_i_ = 9.8 μM), octanoyl-CoA (*K*_*i*_ = 17.0 μM), hexanoyl-CoA (*K*_i_ = 30 μM), butyryl-CoA (*K*_i_ = 190 μM), and Ac-CoA (*K*_i_ = 175 μM) all inhibited AACS noncompetitively (38). The *K*_*i*_s for palmitoyl-CoA and octanoyl-CoA were well below their critical micelle concentrations, thus the inhibition is probably not because of the detergent properties of these compounds (38). Free palmitate did not inhibit AACS. Regulation of AACS by long-chain acyl-CoAs could be a method for feedback regulation of the contribution that AACS can make to denovo fatty acid synthesis. Also, if cytosolic Ac-CoA is in abundance produced from citrate by ACLY, this could affect the flux through AACS as high concentrations of Ac-CoA inhibit AACS (38).

AACS mRNA is found in mouse bone osteoclasts and induced by obesity and/or IL-6 (39). Obesity was also found to affect expression of rat AACS mRNA in skeletal muscle (40). AACS may be involved in glucose-stimulated pancreatic insulin secretion (41, 42, 43, 44). Cytosolic short-chain acyl-CoAs have been hypothesized to have a role in insulin secretion pathways, and knocking down ACLY 80% had no effect on glucose-stimulated insulin secretion while knocking down AACS led to a proportional decrease in glucose-stimulated insulin release (41, 42, 43, 44).

The aforementioned review of AACS demonstrates that it is a highly regulated and high-affinity enzyme involved in lipid synthesis in numerous lipogenic tissues. When upregulated, AACS can generate generous amounts of cytosolic AcAc-CoA and subsequently cytosolic Ac-CoA for denovo lipid synthesis. Its regulation seems closely tied to cholesterol synthesis in some cases but in adipose tissue to fatty acid synthesis. The studies suggest that gene expression of the enzyme can be highly regulated, but other studies, particularly that of Ito *et al.* (5), also suggest that the enzyme may exist in different states with more or less activity. This finding along with the regulation of AACS by acyl-CoAs suggests that there may be much about the regulation of this enzyme that has yet to be described. What is the mechanism for increasing rat hepatic activity 10-fold in 12 h and then decreasing it 10-fold again in the next 12 h; gene expression, activations/deactivations, degradation, allosteric factors? There are other tissues, such as skin, spinal cord, lung, and intestines, that studies on the incorporation of KB into lipids, which suggest that the role of AACS in these tissues should be investigated, but AACS has not been studied in them. Is AACS hormonally regulated? What is unclear is the role of AACS in lipid synthesis in conjunction with ACLY. Both enzymes can generate significant amounts of cytosolic Ac-CoA and AcAc-CoA for denovo lipid synthesis. When and where does each enzyme contribute, what is the role of each, are there situations that upregulate one while downregulating the other? Studies that have been done on KBs as substrates for lipid synthesis may help in answering some of these questions.

## Studies on KB utilization for lipid synthesis

The two metabolic KBs are AcAc and beta-hydroxybutyrate (BHB). They are well recognized as catabolic substances whose concentration rises in fasting, with high-fat diets and in diabetes, and they are used for energy production. These two KBs can readily be oxidized by most cells by a mitochondrial pathway involving mitochondrial 3-oxoacid-CoA transferase, mitochondrial thiolase, and the citric acid cycle (20). A third KB is acetone. It arises via a spontaneous chemical decarboxylation of AcAc and is a waste product not involved in metabolism. In humans, rodents, dogs, and probably most mammals, the two metabolic KBs are present in circulation with minimum total levels around 100–200 μM (45, 46). They are present at all times and with all diets, not just in fasting, diabetes, and with high-fat diets. In fed adult rats (standard rat chows are carbohydrate-rich low-fat diets), Bates *et al.* (45) found AcAc and BHB plasma concentrations of 86 and 95 μM, respectively, and BHB was very rapidly turning over with a half-life of 3.4 min. This implies that KBs are being actively synthesized as well as being actively utilized in the fed state (even when fed a carbohydrate-rich diet), although in lower amounts than in starvation, fat feeding, and diabetes. As was noted earlier, the *K*_*m*_ of AACS for AcAc is quite low, and in virtually any situation, the supply of AcAc should not limit its utilization for denovo lipid synthesis via AACS. It follows that the utilization of KBs as substrates for lipid synthesis, an anabolic process, could be occurring in many situations such as the fed animal, not just situations where KB concentration rises such as in fasting, diabetes, or with high-fat diets (8). The anabolic role of the KBs as substrates for denovo lipid synthesis is a role that is probably not directly associated with or metabolically tied to their catabolic role as energy substrates. The anabolic role of KBs may occur with different diets and in different tissues than the catabolic role. Because AcAc is always present at concentrations to saturate or nearly saturate AACS, the use of AcAc as an anabolic substrate is controlled not by the level of AcAc production but by the regulation of AACS expression and activity.

There are two pathways where the KBs can be incorporated into lipids, a mitochondrial pathway involving 3-oxoacid-CoA transferase, thiolase, synthesis of citrate, citrate transport into the cytosol and ACYL activity to produce cytosolic Ac-CoA, and the cytosolic pathway through AACS. In some studies, hydroxycitrate, an inhibitor of ACYL, has been used to differentiate incorporation through the two pathways.

There are several situations where ^14^C-labeled KBs have been demonstrated to be incorporated into lipids and used for the denovo synthesis of lipids. The two most studied are *1*) lipid synthesis in the infant while nursing and *2*) lipid synthesis from KBs in the adult liver. These two situations will be reviewed first before other tissues and situations are discussed.

### KB utilization for lipid synthesis in the nursing infant

Numerous in vivo studies in rats have demonstrated that while nursing, rats utilize KBs as substrates for lipid synthesis in a variety of tissues (47, 48, 49, 50, 51, 52, 53, 54, 55, 56, 57, 58, 59, 60, 61, 62, 63, 64). A summary of these studies and studies on AACS activity in the suckling animal are presented in Table 2. Much of the focus of these studies has been on KBs being utilized as substrates for lipid synthesis in the developing rat brain (47, 48, 49, 50, 51, 52, 53, 54, 55, 56, 57, 58, 59, 60, 61). During the suckling period in rats, brain development and myelination are occurring, and the brain is an active lipogenic tissue with denovo synthesis of sterols and fatty acids occurring as well as synthesis of other lipids such as phospholipids and sphingolipids. These lipids are needed for the process of myelination as well as other processes that are very active during early brain development. The in vivo studies of Edmond (47) and Webber and Edmond (48, 49) on the incorporation of radio-labeled KBs in suckling rats demonstrated incorporation of KBs into brain lipids. The KBs were incorporated much better than labeled acetate or glucose, and the label was preferentially incorporated into cholesterol (47, 48, 49). In vivo studies on brain regions from Yeh *et al.* (51, 53) showed that during active periods of myelination (12–20 days of age in the rats), incorporation of labeled [3-^14^C]AcAc into lipids was highest in brain stem and then next highest in cerebrum and thalamus. The pattern of incorporation of ^14^C-AcAc coincided with evidence that active myelination begins in the hindbrain and proceeds rostrally toward the forebrain. The changes in rates of incorporation of AcAc into lipids during development were closely related to AACS activity but not to ACLY or Ac-CoA synthetase activities (53). Whole brain homogenates (52, 60) and cerebral cortex brain slices (61) from suckling rats readily incorporate ^14^C-KBs into lipids. Whole brain homogenates synthesized lipids from AcAc at a rate that was 7–11 times higher than the rate lipids were synthesized from glucose (52). It was concluded that AcAc and BHB were preferred over glucose as substrates for the synthesis of lipids in brain of these suckling animals (52). The high rate of lipid synthesis from the KBs was accompanied by increased activity of AACS (52).

**Table 2:** Findings from AACS studies and KB to lipid studies in suckling animals and rat fetus

Tissue	AACS	KB to lipids
Suckling animal		
Brain (rat)	• AACS is high in suckling period (2) • AACS activity changes during development correlated with AcAc incorporation into lipids during brain development (52)	• In vivo, KBs readily incorporate and preferentially label sterols; incorporation is much better than acetate or glucose (47, 48, 49, 50, 51, 52, 53, 54, 55, 56, 57, 58, 59, 60, 61) • KBs preferred substrates for lipid synthesis (47, 48)
Brain homogenates	• KBs to lipids is accompanied by increases in AACS (52)	• AcAc 7–11 times is better than glucose as a substrate for lipid synthesis (52)
Brain regions		• Highest in brain stem > cerebrum > thalamus, coincides with active myelination (51, 53)
Oligodendrocytes (calf)	• AACS is present (65)	• AcAc to lipids is not inhibited by hydroxycitrate (65)
Oligodendrocytes (suckling rat)		• KBs are readily used for energy and as substrates for lipid synthesis (59) • Glucose was a poor substrate for lipid synthesis (59)
Mixed cultures of oligos and astrocytes		• AcAc is a better substrate for lipid synthesis than glucose by a factor of 5–10 (57) • Glucose stimulates AcAc incorporation into lipids, whereas AcAc reduces the entry of glucose into lipids (57)
Spinal cord (suckling rat)		• KBs readily incorporated and preferentially label sterols (47, 48, 56, 66)
Skin (suckling rat)		• In vivo, KBs readily incorporated and preferentially label sterols (47, 48)
Lung (suckling rat)		• KBs to lipid highest at birth decreases 5-fold to adult levels by day 5 (62) • Regardless of age, AcAc was a better substrate for lipid synthesis than glucose (50, 62, 63, 64, 67, 68) • Hydroxycitrate blocks glucose incorporation into lipids but not AcAc incorporation (64)
Developing fetus		
Rat fetus at 21st day of pregnancy		Maternal KBs cross placental barrier and are incorporated into lipids into fetal tissues, including brown adipose tissue, pancreas, liver, and lung (69)

Oligodendrocytes in the developing brain are the cells that are principally synthesizing myelin. The KBs as substrates for lipid synthesis have been studied in cultured oligodendrocytes prepared from the brains of suckling rats (59), mixed suckling rat oligodendrocyte and astrocyte cultures (57), and oligodendrocyte cultures isolated from calf brain (65). Sykes *et al.* (59) found that glucose was a relatively poor substrate for lipid synthesis and that the KBs were used as energy sources and precursors for the denovo synthesis of cholesterol and fatty acids in the rat oligodendrocytes. Koper *et al.* (57) found that in both astrocytes and oligodendrocytes, AcAc was a better precursor for lipid synthesis than was glucose by a factor of 5–10. Glucose stimulates AcAc incorporation into lipids (probably by helping with NADPH production), whereas AcAc reduces the entry of glucose into lipids (57). Pleasure *et al.* (65) found AACS activity in calf oligodendrocytes, and evidence with the incorporation of [3-^14^C]AcAc into lipids and the effects of hydroxycitrate showed that AcAc is actively being incorporated into lipids in calf oligodendrocytes via an extramitochondrial pathway (the AACS pathway). In addition to the brain, the spinal cord is undergoing myelination during the suckling period in the rat. Studies by Edmond (47), Webber and Edmond (48), Lopes-Cordozo *et al.* (56), and Ramsey (66) all demonstrate that radiolabeled KBs are readily utilized for lipid synthesis in the spinal cord. When suckling 18-day-old rats are subcutaneously injected with ^14^C-labeled AcAc or BHB, the spinal cord incorporated more label into sterols and fatty acid per gram tissue than did brain, skin, kidney, or liver (47, 48). Lopes-Cordozo *et al.* (56) found with ^3^H_2_O that in 21-day-old rats, spinal cord was synthesizing twice as much fatty acids and 3-fold more sterols than was whole brain on a per gram basis. Spinal cord oligodendrocytes are responsible for synthesizing spinal cord myelin. These data suggest that spinal cord and its oligodendrocytes are particularly active in using KBs as substrates for lipid synthesis.

AACS and KB utilization in adult rat brain appears to be quite different from that in suckling animals. Ohnuki *et al.* (70) examined by in situ hybridization the localization of AACS mRNA in rat brain from adult male rats about 50 days old, and the pattern appears to be quite different from that in suckling animals. The labeling in the adult male rats was high in the midbrain, pons/medulla, cerebral cortex, hippocampus, and cerebellum. While in the suckling animals, KB incorporation into lipids was highest in brain stem and then next highest in cerebrum and thalamus (51, 53).

### KB utilization for lipid synthesis in other tissues of the nursing infant

Evidence that skin of suckling rats can utilize the KBs as substrates for lipid synthesis comes from the initial studies of Edmond (47) and Webber and Edmond (48, 49). These were in vivo studies on 9–12-day-old rats (47) and on 18-day-old rats (48, 49). In these studies, labeled precursors, including AcAc, BHB, acetate, mevalonate, and glucose, were injected subcutaneously, and incorporation of label into lipids of various tissues was studied. Label from AcAc and BHB was readily incorporated into lipids from spinal cord, brain, and skin, all tissues of ectodermal origin (47, 48, 49). The label in these three tissues was much greater than that found in lipids in kidney, lung, liver, and blood.

The studies of Yeh *et al.* on the incorporation of KBs into lung surfactant lipids during the early suckling period of rats show that this process of lipid synthesis from KBs is also ongoing in the lung (50, 62, 63, 64, 67, 68). Infants require lung surfactant lipids in order to breath. Sheehan and Yeh (62) studied lipid synthesis in minced lung tissue from rats at birth, through the sucking period, and in adults. Total lung lipid synthesis is highest at birth and rapidly decreases about 5-fold so that by day 5, it approaches the low level found in adults. Studies with [3-^14^C]AcAc and [U-^14^C]glucose showed that in all animals, regardless of age, AcAc was a better substrate for lipid synthesis and energy production than glucose in the lung. Glucose was readily incorporated into the glycerol backbone of lipids but not so readily into the FA and sterol components. Hydroxycitrate inhibited glucose incorporation into lipids by 88% but did not affect the incorporation from AcAc showing that the AACS and cytosolic thiolase pathway is responsible for major route for denovo fatty acid synthesis in the lungs of developing rat (64).

The studies cited above demonstrated that the KBs are major substrates for the denovo synthesis of lipids in several tissues of suckling rats, including brain, spinal cord, skin, and lung. In these tissues, the KBs serve as much better substrates for lipid synthesis than does glucose, and the incorporation of the KBs is largely through an extra mitochondrial pathway via AACS.

### Lipid synthesis from KBs in adult liver

The utilization of KBs for lipid synthesis has been studied in perfused rat livers from fed and fasted animals (71) and from streptozocin-induced diabetic rats (72), in isolated rat hepatocytes (73), and in vivo in adult rats (4). A summary of studies on AACS and KB utilization for lipid synthesis in adult tissues is presented in Table 3. Endemann *et al.* (71) found that in perfused livers, KBs contribute 19–80% of the carbon for sterol synthesis and up to 22% of the carbon for FA synthesis, depending on the metabolic status. Freed *et al.* (72) found that streptozocin-induced diabetes decreased the total lipid synthesis by 80–95% and AACS was decreased by 17%, and the incorporation of KBs into lipids was markedly inhibited by streptozocin-induced diabetes. Geelen *et al.* (73) studied lipid synthesis with ^3^H_2_O and [3-^14^C]AcAc in isolated rat hepatocytes from meal-fed, fed, starved, lactating, and starved lactating rats. They found that the contribution from AcAc varied from 14 to 54% for denovo FA synthesis and from 21 to 75% for denovo cholesterol synthesis depending on the physiological condition of the donor rat. The highest percent contribution of AcAc to lipid synthesis occurred in the fed and lactating animals, the animals with the highest rates of overall lipid synthesis, and in both cases, animals were fed with rat chow, a carbohydrate-rich diet. In hepatocytes from the fed animals, 36% of the total synthesis of FAs and 60% of the total synthesis of cholesterol came from AcAc, and in hepatocytes from the lactating animals, 36% of the total carbon going for FA synthesis and 72% of cholesterol synthesis came from AcAc (73). Bergstrom *et al.* (4) found that AcAc was used in vivo preferentially for cholesterol synthesis when compared with acetate or glucose. These studies demonstrate that the KBs can be significant substrates for both fatty acid and cholesterol synthesis in rat liver, and the contribution can vary depending on the state and diet of the animal. The studies show that the KBs can be readily used in liver, and there is a preferential utilization of AcAc as a substrate for cholesterol synthesis over FA synthesis. The mitochondrial pathway for KB utilization involving 3-oxoacid-CoA transferase is absent in liver, thus KB utilization in liver is via AACS, and this utilization is for denovo lipid synthesis. AcAc was readily used via AACS for lipid synthesis in liver in these experiments where rats were fed a carbohydrate-rich diet. This emphasizes the importance of this pathway in the fed state.

**Table 3:** Findings from AACS studies and KB to lipid studies in adult animals and adult human

Tissue	AACS	KB to lipids
Adult		
Liver (rat)	• Low *K*_*m*_, high affinity for AcAc (3, 8, 28) • Low infant, high adults (2, 3, 27) • 10-fold diurnal variation (3) • Meal feeding increases (4, 8, 29) • Fasting decreases (3, 5, 6) • Regulated by cholesterol, cholestyramine, mevalonolactone, and statins (4, 5, 30) • Olive oil feeding increases (33) • SREBP-2 in promoter region (18) • Higher in females than males (27) • Perfusion of liver with oleate increased AACS activity (32) • AACS knockdown in mice lowers serum cholesterol (18) • Acyl-CoAs such as palmitoyl-CoA are low micromolar noncompetitive inhibitors (38)	• Perfused liver from fed, fasted, or streptozocin-diabetes o KBs contribute 19–80% of carbon for sterol synthesis o KBs contribute up to 22% of carbon for FA synthesis o KBs to lipid markedly inhibited by streptozocin • Hepatocytes from meal-fed, fed, starved, lactating, and starved lactating rats o AcAc contributed 14–54% of carbon for FAs o AcAc contributed 21–75% of carbon for cholesterol o Highest rates were for fed and lactating animals o AcAc preferentially utilized for cholesterol synthesis • In vivo, KBs preferentially utilized for cholesterol synthesis (4)
Adipose tissue (AT)	• AACS activity is higher in adult female AT than adult liver and brain (5) • mRNA is abundant in white AT of male rats after weaning (35) • Inducers of adipocyte differentiation increased mRNA for AACS and ACC-1 (9, 18, 19, 35) • AACS promoter region has a C/EBP-binding site, and expression is controlled by C/EBPa during adipocyte differentiation (9) • AACS promoter region contains a binding site for PPARg, a regulator of adipocyte differentiation (19)	• AcAc is an important substrate for lipid synthesis (74, 75, 76) • Insulin and glucose stimulate AcAc utilization for lipid synthesis (75) • AcAc is readily used for FA synthesis (74, 75, 76)
Mammary gland (rat) (in vivo, slices, acini)	• High during lactation, low before and after weaning (2)	• In vivo, BHB is incorporated into lipids (7) o 24% of whole-body turnover of BHB is directed toward mammary gland lipid synthesis (7) • 2 mM AcAc reduced glucose incorporation into lipids by 54% in slices and 89% in acini (14, 77) • Insulin increased AcAc incorporation into lipids in slices
Intestine		• 24% of whole-body denovo cholesterol synthesis occurs in the intestines (78) • Rat colonic epithelial cells (79) o Data suggest that AcAc is a major substrate for lipid synthesis in these cells (79) o Citrate was utilized for lipid synthesis “to a limited extent if at all” (79) o Acetate and butyrate are also used for lipid synthesis (79) • Neonatal chicken mucosa uses AcAc for lipid synthesis via AACS (80)
Skin		• Human diploid fibroblasts (16) o AcAc is a better substrate for lipid synthesis than glucose, glutamine, lactate, and pyruvate by at least a factor of 2 (16) o AcAc was 7.7 times better than glucose for sterol synthesis (16) o Apparent *K*_*m*_ for AcAc of 30 and 185 mM for cholesterol and FA synthesis, respectively (16) o Hydroxycitrate blocks pyruvate to lipids by not AcAc (16) o LDL reduces AcAc to sterols by 56% and to FAs by 75% (16)
Adrenals (rat)	• Regulated in conjunction with HMG-CoA reductase (4) • 4-APP treatment induces activity 7.3-fold (4)	
Bone osteoclasts (mouse)	• mRNA is upregulated with high-fat diet (39)	
Adult brain capillaries (rat)		• KBs readily incorporated into lipids, glucose label in lipids mainly in glycerol part of phospholipids (54)
Adult brain microvessel endothelial cells (rat brain)		• KBs, especially AcAc, are preferred substrates for lipid synthesis (54)
Murine sciatic nerve		• KBs are well utilized as substrates for lipid synthesis, preferentially for sterol synthesis (81)

### KB utilization for lipid synthesis in other tissues

KB utilization for lipid synthesis has also been studied in rat and mouse adipose tissue (11, 74, 75, 76), rat lactating mammary gland (7, 14, 77), isolated adult rat brain capillaries (54), adult rat brain microvessel endothelial cells (55), glioma C6 and neuroblastoma C1300 cells (82), four neural cell lines (83), rat colonic epithelial cells (79), skin (47, 48), human skin diploid fibroblasts (16), pancreatic islet cells (41, 42, 43, 44), isolated rat hepatoma cells (15), and rat fetus (69).

Rous and Favarger (74) found an important role for AcAc in producing cytosolic Ac-CoA in adipose tissue of mice and rats. Hanson and Ziporin (75) found that ^14^C-labeled BHB or AcAc was well incorporated into lipids by mouse epididymal fat pads, and glucose, insulin, and nicotinamide stimulated their utilization for lipid synthesis. Yoo *et al.* (76) found that in brown adipocytes in the presence of 10 mM AcAc, 70% of lipid synthesis came from AcAc, decreasing the contribution from glucose and glutamine to 30%, which included the portion from glucose in the glycerol backbone of the triglycerides. These studies along with the studies discussed previously on the expression and regulation of AACS in adipose tissue suggest that this is a tissue where KBs can readily be used as substrates for fatty acid synthesis.

KB utilization for lipid synthesis and their influence on glucose utilization for lipid synthesis has been studied in rat mammary gland, mammary gland slices, and acini isolated from mammary gland, all from lactating female rats (7, 14, 77). Mammary gland was found to incorporate labeled BHB into mammary gland lipids (7). In the in vivo experiments, fed lactating rats incorporated into mammary gland lipids as much as 20% of the BHB dose given to the whole animal suggesting that the KBs are making a significant contribution to mammary gland lipid synthesis (7). The blood KB level was 0.15 mM in these fed lactating animals (7). Both labeled AcAc and labeled BHB were found to be incorporated into lipids by acini (77), and labeled AcAc was incorporated into lipids by mammary gland slices (14). AcAc, 2 mM, reduced [1-^14^C]glucose utilization for lipid synthesis by slices by 34% (14). AcAc (2 mM) added to slices (14) or acini (77) decreased the incorporation of [6–^14^C]glucose into lipid by 54% and 89%, respectively. Insulin increased the incorporation of AcAc into lipids in mammary gland slices (14) and mammary gland acini (77). The results demonstrated that mammary gland from lactating rats readily use KBs as substrates for lipid synthesis in the fed animal when KBs are at a fairly low concentration (0.15 mM) in circulation, and they can decrease the utilization of glucose for lipid synthesis.

### KB utilization for lipid synthesis in the skin

The skin is the largest organ of the body, and it has been estimated that cholesterol synthesis in skin represents 12% of the body’s total denovo cholesterol synthesis in rat, 26% in the squirrel monkey, 19% in the hamster, 20% in the rabbit, and 18% in the guinea pig (84). Cholesterol synthesis in skin is required to maintain the water permeability barrier that skin provides (85). Studies in the suckling rat by Edmond (47) and Webber and Edmond (48) demonstrated that ^14^C-labeled KBs were well incorporated into skin lipids. The utilization of KBs for lipid synthesis has also been studied in skin diploid human fibroblasts (DHFs) (16). Incorporations of ^14^C from AcAc, D(−)-BHB, L(+)-BHB, glucose, glutamine, lactate, and pyruvate into lipids were studied in cultured DHFs. AcAc was a better substrate for lipid synthesis than any of the other substrates including glucose by 2- to 8-fold. The *K*_*m*_ apparent for AcAc incorporation in sterols and FAs was 30 μM and 185 μM, respectively, showing that AcAc was incorporated at low concentrations. Hydroxycitrate, an ACLY inhibitor, in DHF inhibited the incorporation of ^14^C from pyruvate by 99% but did not affect the incorporation of ^14^C from AcAc, demonstrating the AcAc incorporation does not go through ACLY. Glucose stimulated AcAc incorporation into lipids, and conversely, AcAc inhibited glucose incorporation into lipids. In the presence of AcAc and glucose, digitonin precipitable sterols were labeled from ^14^C-AcAc 7.7 times better than from ^14^C-glucose. The presence of LDL inhibited AcAc incorporation into sterols by 56%, whereas it inhibited glucose incorporation by 75% (16). The results from these studies suggest that some cells in skin, both rat and human, can readily utilize the KBs as substrates for denovo lipid synthesis, that AcAc is a much better substrate for lipid synthesis than is glucose in these cells, and that the pathway for AcAc utilization bypasses ACLY. The cells that are major contributors to skin lipid synthesis include sebaceous glands, hair follicles, and keratinocytes. It would be of interest to study KB utilization for lipid synthesis in these skin cells, particularly keratinocytes that are responsible for establishing and maintaining the water permeability barrier of skin (85).

### KB utilization for lipid synthesis in intestinal cells

Epithelial cells of the intestines are one of the most rapidly turning over cells of the body, and they require phospholipids and cholesterol for formation of the newly created plasma membranes. It has been estimated that in rats, intestinal mucosa synthesizes the majority (64–86%) of its cholesterol rather getting it delivered by lipoproteins (80). Zambell *et al.* (79) investigated what substrates are used by rat colonic epithelial cells and found that acetate, butyrate, and KBs were well utilized for lipid synthesis, and conversely, citrate was used to a limited extent if at all. In the rat, the intestinal mucosa accounts for 24% of the whole body’s denovo cholesterol synthesis (78). The study by Zambell *et al.* (79) with rat colonic epithelial cells suggests that AcAc is probably a major substrate for the synthesis of lipids in this organ that synthesizes a significant portion of the body’s cholesterol. KBs were also found to be utilized for lipid synthesis in neonatal chicken duodenal mucosa, and the evidence suggested that AcAc was utilized directly via AACS (80).

### KB utilization for lipid synthesis in adult nervous cells and tissues

Rous and Favarger (74) studied lipid synthesis in cultured rat brain microvessel endothelial cells. The KBs, especially AcAc, were the preferred substrates for lipid synthesis. Energy production and lipid synthesis from the KBs were studied in isolated adult rat brain capillaries (54). The KBs were incorporated mainly into not only phospholipids but also labeled sterols. [U-^14^C]Glucose heavily labeled phospholipids, but the label was almost exclusively in the glycerol backbone. Clouet and Bourre (81) found that KBs were well utilized for lipid synthesis in murine sciatic nerve. The incorporation into sterols was greater than into other lipids.

### KB utilization for lipid synthesis in cancer and permanent and cancerous cell lines

A summary of studies on AACS and KB utilization for lipid synthesis in permanent and cancerous cell lines is shown in Table 4. Roeder *et al.* (83) studied the utilization of KBs and glucose by four established neural cell lines, neuroblastoma of rat (B103), neuroblastoma of mouse (N4TG1), rat astrocytoma (RGC6), and mouse oligodendroglia (G2620). In all four cell lines, the incorporation of AcAc into lipid was three to five times higher than from glucose. The rates of oxidation in three of the four lines were highest for glucose. It was concluded that the KB utilization was directed toward lipid synthesis, whereas glucose was the preferred energy source for these cell lines (83). Patel *et al.* (82) studied KB metabolism in rat glioma C6 cells and mouse neuroblastoma C1300 (N2a) clones. The KBs, especially AcAc. were the preferred substrates for lipid synthesis in both cell lines, and the incorporation of glucose into lipids was significantly reduced in the presence of the KBs.

**Table 4:** Findings from AACS studies and KB to lipid studies in permanent cell lines and cancer cells

Tissue	AACS	KB to lipids
Cell lines		
Rat Morris hepatoma 7777 cells freshly isolated from malignant tumors		• AcAc, 8.4-fold, and BHB, 17-fold, were better substrates for cholesterol synthesis than was glucose (15) • AcAc, 4.0-fold, and BHB, 7.9-fold, were better substrates for FA synthesis than was glucose (15) • Hydroxycitrate reduced incorporation from pyruvate 99%, BHB 43%, and enhanced incorporation from AcAc by 2% (15)
Neuroblastoma C1300		• AcAc is the preferred substrate for lipids, and the presence of AcAc reduced the incorporation of glucose into lipids (82)
Glioma C6		• AcAc is the preferred substrate for lipids, and the presence of AcAc reduced the incorporation of glucose into lipids (82)
Neuroblastoma rat B103		• AcAc to lipids is 3–5 times better than glucose, BHB to lipids about equal to glucose. Glucose was the preferred oxidative substrate (83)
Neuroblastoma mouse N4TG1		• AcAc to lipids is 3–5 times better than glucose, BHB to lipids about equal to glucose. Glucose was the preferred oxidative substrate (83)
Rat astrocytoma RGC6		• AcAc to lipids is 3–5 times better than glucose, BHB to lipids about equal to glucose. Glucose was the preferred oxidative substrate (83)
Mouse oligodendroglia G2620		• AcAc to lipids is 3–5 times better than glucose, BHB to lipids about equal to glucose. Glucose was the preferred oxidative substrate (83)

Hildebrandt *et al.* (15) studied KB utilization for lipid synthesis in Morris hepatoma 7777 cells freshly isolated from highly malignant tumors grown in the hindlimb of buffalo rats. The relative rates compared with glucose incorporation into cholesterol were 17.0 for BHB, 8.4 for AcAc, 1.9 for pyruvate, and 1.0 for glucose. Hydroxycitrate reduced by 99% the incorporation from pyruvate, by 43% from BHB, and enhanced incorporation by 2% for AcAc. FAs were also synthesized from BHB (7.9-fold better than glucose) and AcAc (4.0-fold better than glucose). Hepatocytes isolated from the host animal also incorporated glucose in the cholesterol and FAs but at rates that were 2-fold to 3-fold less than the malignant cells. The host hepatocytes used AcAc but at rates about 3X and 2X of glucose for cholesterol and FA synthesis, respectively. The use of KBs for the synthesis of lipids was greatly enhanced in these highly malignant hepatoma cells as compared with the host hepatocytes, and the utilization of the KBs for lipid synthesis was much greater than the utilization of glucose. AcAc was used by a pathway that was not inhibited by hydroxycitrate, whereas some of the utilization of BHB was inhibited by hydroxycitrate (15). Hildebrandt *et al.* (86) in a second study found that the cytosol of the Morris hepatoma 7777 cells contained an activity where D(−)-BHB was activated to cytosolic 3-hydroxybutryl-CoA, an enzyme activity that has not been described elsewhere. In these last two paragraphs of this review, seven different cancerous cell lines have been discussed that have all readily used the KBs for lipid synthesis and at rates many fold higher than the rates they used glucose for lipid synthesis.

### KB utilization for lipid synthesis in the fetus

Secombe *et al.* (69) studied KB utilization for lipid synthesis in rat fetuses. [3–^14^C]BHB was injected into the femoral vein of pregnant female Sprague-Dawley rats on their 21st day of gestation (birth occurs on day 22). At 5 min postinjection, labeled BHB was found in the blood of the fetuses demonstrating that the KB can cross the placental barrier. Label was then rapidly incorporated in phospholipids, acylglycerols, and cholesterol in a variety of fetal tissues. Incorporation was greatest in brown adipose tissue followed by pancreas, liver, and lung. Experiments where the fetuses were directly injected in utero confirmed that the BHB was used directly by the fetus for lipid synthesis (69). This is a case where KBs, made by the mother, are transferred to the fetuses, and they are then used by the fetuses as a substrate for lipid synthesis.

### Preferential utilization of AcAc for cholesterol synthesis and the channeling of AcAc into cholesterol as a 4-carbon unit

Several lines of evidence suggest that the KBs via AACS can have a preferential role in cholesterol biosynthesis and that there can be substrate channeling of AcAc directly into cholesterol (isoprenoid) synthesis bypassing the cytosolic Ac-CoA pool. Webber and Edmond (49) injected ^14^C-labeled acetate, glucose, AcAc, or BHB into 18-day-old rats and determined the ratio of ^14^C going into sterols/fatty acids (Table 5). AcAc and BHB produced ratios for label in sterols/label in fatty acids that were higher than those for acetate and glucose. This was interpreted as evidence that some of the AcAc was going directly into making HMG-CoA and then cholesterol as a 4-carbon unit rather than first being cleaved by thiolase to produce Ac-CoA, which was then used as the starting substrate for lipid synthesis (49). This preferential incorporation of AcAc into sterols has also been observed in an in vivo study in adult rats (4), isolated rat hepatocytes (73), perfused rat liver (71), isolated rat hepatoma cells (15), in the synthesis of myelin in suckling rats (58), suckling rat brain and spinal cord (56), and in human skin diploid fibroblasts (16).

**Table 5:** Preferential utilization of KBs for synthesis of sterols in 18-day-old rat brain (49) and in adult rat liver (4)

Substrate injected in vivo	18-Day-old rat brain	Adult rat liver
	Webber and Edmond (48)	Bergstrom *et al.* (4)

	Ratio dpm in sterols per gram/dpm in fatty acids per gram	

d-(−)-3-hydroxy[3–^14^C]butyrate	0.475 ± 0.015	
[3-^14^C]Acetoacetate	0.494 ± 0.015	0.30
[2-^14^C]Glucose	0.225 ± 0.014	0.13
[2-^14^C]Acetate or [1-^14^C]acetate	0.296 ± 0.016	0.13
[1-^14^C]Octanoate	0.289 ± 0.016	

Data are from Table 3 from Webber and Edmond (48) and Table 1 of Bergstrom *et al.* (4). Animals were injected subcutaneously with the above labeled substrates and dpm in sterols/fatty acids determined brain or liver as described (4, 49).

A ^13^C study by Miziorko *et al.* (87) demonstrated the channeling of AcAc as a 4-carbon unit into sterol synthesis. Adult female rat liver and brain cytosol were incubated with [1-^13^C]acetate or [3-^13^C]AcAc and lovastatin and other cofactors. Cytosolic HMG-CoA would accumulate in these incubations because of the inhibition of HMG-CoA reductase by lovastatin. The incubations were extracted, base hydrolyzed to remove CoA esters, acids extracted and methylated, and then GC/MS used to determine the enrichment of ^13^C in HMG. [1-^13^C]Acetate gave a triple labeling pattern that labeled the 1, 3, and 5 positions of HMG, whereas [3-^13^C]AcAc predominantly gave HMG that was monolabeled in the 3-position (87). This shows that AcAc was predominately incorporated into HMG as a 4-carbon unit. That AcAc-CoA would be channeled directly into the synthesis of HMG-CoA is understandable because of the kinetics of the enzymes involved (4). Cytosolic HMG-CoA synthase is an enzyme with high affinity for AcAc-CoA (*K*_*m*_ < 2 μM), whereas cytosolic thiolase has a lower affinity for AcAc-CoA (*K*_*m*_ = 50 μM). As AcAc-CoA is produced by AACS, it can be utilized directly for cholesterol synthesis by HMG-CoA synthase, the high-affinity enzyme prior to its being cleaved by thiolase. A special role for AcAc in cholesterol synthesis in some situations is supported by the regulation of AACS by cholesterol and the SREBP regulatory element in its promoter.

### Conclusions: KB utilization for denovo lipid synthesis

From this section on the utilization of KB for lipid synthesis, the following conclusions can be made.

1 .The anabolic role of the KBs as substrates for denovo lipid synthesis can occur in just about any metabolic situation where lipid synthesis is occurring as AACS is a high-affinity enzyme and the AcAc concentration even in the fed state (including on a carbohydrate-rich diet) is high enough to supply KBs for the utilization for denovo lipid synthesis;
2 While the rat is nursing, the KBs serve as the most important precursors for denovo lipid synthesis in numerous organs including the brain, spinal cord, skin, and lung, all tissues with active lipid synthesis during this period of early development. The KBs are utilized for lipid synthesis at rates several fold higher than is glucose in these tissues during the suckling period;
3 AcAc can be utilized by the liver for lipid synthesis and particularly for cholesterol synthesis. The metabolic state of the animal and the data with oleate perfusions and olive oil feeding suggests that diet and other factors may affect the part the AcAc plays in lipid synthesis in the liver;
4 Mammary gland and adipose tissue do utilize KBs for lipid synthesis, the presence of AcAc depresses glucose utilization for lipid synthesis in mammary gland;
5 Preliminary evidence suggests that numerous other tissues, many with active cholesterol and lipid synthesis such as skin and intestinal mucosa, can readily use KBs for lipid synthesis and particularly for cholesterol synthesis. The evidence suggests that AcAc was a much better substrate in some cells in these tissues for lipid synthesis than was glucose;
6 Studies in permanent cell lines and freshly isolated Morris hepatoma 777 cells found that AcAc is utilized for lipid synthesis much better (3-10-fold better) than is glucose, and in the presence of AcAc, glucose utilization for lipid synthesis is suppressed;
7 The developing fetus utilizes KBs supplied by its mother for lipid synthesis;
8 The KBs are a preferred substrate for cholesterol synthesis, and they can be incorporated via a high-affinity pathway directly into cholesterol as a 4-carbon unit.

## Physiological roles for AACS and KBs as substrates for denovo lipid synthesis

AACS, a high-affinity enzyme, and the process of KB utilization for lipid synthesis is a high-affinity process that only requires a low concentration of AcAc. The *K*_*m*_ of AACS is low micromolar, and the apparent *K*_*m*_ for the process of AcAc to cholesterol in cultured cells is also low micromolar. These concentrations of AcAc and KBs are present in virtually all metabolic situations including the fed state, thus substrate supply does not drive the anabolic process of the utilization of the KBs for lipid synthesis. This anabolic role of KBs is probably most important in normal-fed states and tissues, not in situations like starvation and diabetic ketoacidosis where KB concentrations rise to high levels but where little lipid synthesis is occurring. This anabolic role for the KBs needs to be considered apart from their catabolic roles. Because substrate supply does not limit this pathway, this suggests that the regulation of AACS activity is critical for determining flux through this pathway. As the review above shows, AACS is a highly regulated lipogenic enzyme, but there may yet be much to find out about how it is regulated and particularly its regulation in conjunction with or in contrast to that of ACLY, the other major enzyme for supplying carbon for denovo lipid synthesis.

AACS and the utilization of KBs for lipid synthesis appear to have a role in cholesterol synthesis in many tissues and cases. AACS is regulated by modulators of cholesterol synthesis, has an SREBP regulatory element, and is regulated in conjunction with other enzymes of cholesterol synthesis including HMG-CoA reductase in many tissues. KBs when compared with other substrates such as glucose and acetate often are preferentially utilized for sterol synthesis over fatty acid synthesis. It has also been demonstrated that AcAc is incorporated into cytosolic HMG-CoA as a 4-carbon unit. AcAc-CoA can be generated by the high-affinity enzyme AACS and utilized directly by another high-affinity enzyme, cytosolic HMG-CoA synthase. Generating AcAc-CoA via the high-affinity enzyme AACS may be an easier path for generating AcAc-CoA than through generation by the low-affinity enzyme AcAc-CoA thiolase, *K*_*m*_ for Ac-CoA of 50 μM (88). Where it has been investigated, AACS or the utilization of KBs for lipid synthesis suggests that this enzyme and process may be important in nonhepatic tissues, such as skin, intestinal mucosa, adrenals, and spinal cord. These tissues are known to actively synthesize much of their own cholesterol rather than getting it from lipoproteins, and they contribute to a significant fraction of total body cholesterol synthesis (84, 89). AACS has not been studied in many of these tissues, including skin, the intestine, spinal cord during infancy, and the lens of the eye. In skin, for instance, whether AACS is there is unknown, and if the water permeability barrier is disrupted, is the activity and expression of AACS changed? (85). The lens of the eye has a very high concentration of cholesterol, and it synthesizes it rather than obtaining it from lipoproteins. Defects in cholesterol synthesis and cholesterol synthesis inhibitors can cause cataracts (90). Are the KBs used for cholesterol synthesis by the lens? AACS is present and regulated in liver, and the KBs can be utilized for lipid synthesis and maybe especially for cholesterol synthesis in liver. Can diet affect the contribution of this process in liver? The results of oleate perfusion and olive oil feeding suggest that this is an area that needs investigation.

### Lipid interconversion

**Fig. 2:**
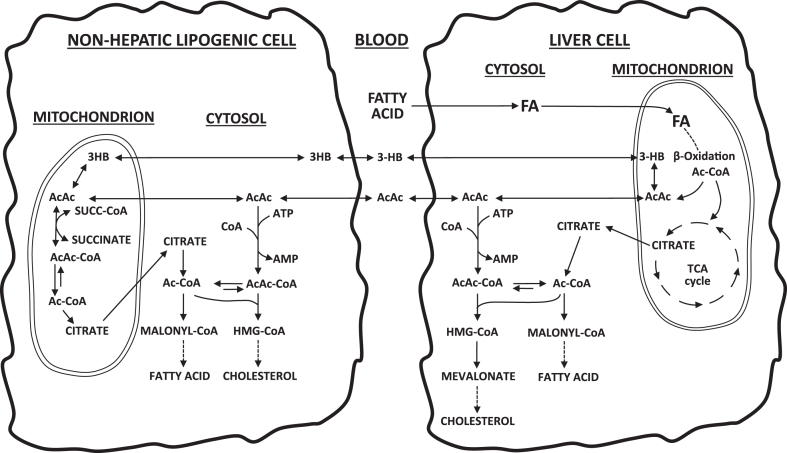
Lipid interconversion pathways. Fatty acids, from the diet or from lipid stores, are oxidized in the liver mitochondria to produce the KBs. AcAc can be utilized in the cytosol of liver via hepatic AACS to produce lipids, that is, cholesterol and fatty acids. AcAc and BHB can also enter the blood where they are transported to other tissues that are making lipids. There AcAc can be utilized for lipid synthesis via AACS. The net result is that dietary or stored fatty acids are converted into other lipids via the KB and AACS.

The review above clearly demonstrates that at times the KBs, with activation through AACS, can be important substrates for denovo lipid synthesis. The KBs are generated from oxidation of FAs in the liver and then can be used for lipid synthesis in liver and other tissues. This is a process of lipid interconversion. The pathways for lipid interconversion, either within the liver or with KBs produced in the liver being utilized for lipid synthesis via AACS in other lipogenic tissues, are depicted in Fig. 2. This leaves the questions of when is this an important process and what does it accomplish metabolically? Possible answers to these questions may come from examining situations where it has been demonstrated to be of importance, for instance, lipid synthesis in a number of tissues during the suckling period and cholesterol synthesis in liver and considering the sources for making the KBs.

The KBs largely are generated from the oxidation of fatty acids in liver and some in kidney. Mitochondrial Ac-CoA generated from FA oxidation can be converted to AcAc and subsequently BHB through the action of the mitochondrial thiolase, mitochondrial HMG-CoA synthase, and then the mitochondrial HMG-CoA lyase, an enzyme expressed in hepatic and renal tissues (20). KBs can also be derived from the catabolism of the ketogenic amino acids, particularly leucine, but in general, KBs mainly arise from the oxidation of FAs (20).

The suckling rat is receiving a diet, milk, that is rich in fat and protein and low in carbohydrate. Fat accounts for 60–70% of its caloric content, and it is rich in saturated FAs with up to 32% of FAs are saturated medium-chain FAs (MCFAs) (91, 92). The MCFAs as well as the long-chain FAs are readily oxidized and can be a source for the generation of KBs (91, 92). The KBs made from the milk FAs are then readily used for energy production and to synthesize lipids. The growing suckling rat requires an abundance of lipids, for instance, every new cell needs cholesterol and phospholipids for its cellular membrane, skin requires cholesterol and other lipids for creating its water impermeability barrier, lungs require surfactant lipids, and the growing brain and nervous system undergoing myelination requires cholesterol, sphingolipids, and phospholipids. The lipids in milk do not necessarily match the needs of the growing infant. Dietary FAs are being used to make lipids in a variety of tissues in the suckling rat. Cholesterol synthesis is important, but other lipids are also being made. This is a process of lipid interconversion; milk FAs converted to cholesterol and other lipids in the suckling animal.

Here, I hypothesize that an example of lipid interconversion likely occurs during the suckling period and involves very long-chain fatty acids (VLCFAs) of the brain sphingolipids. The KBs are derived from the oxidation of FA supplied in the mother’s milk. A group of lipids that are needed but not found in milk are the VLCFAs (20–26 carbons long) needed for sphingolipids during myelination (92). Sphingomyelins, ceramides, and gangliosides of brain and spinal cord contain a high percentage of VLCFAs (up to 50%) (93). The VLCFAs are synthesized in the brain by chain elongation starting with 16 or 18 carbon fatty acyl-CoAs and adding Ac-CoA (not malonyl-CoA) (94, 95). Where does the Ac-CoA for the process of chain elongation come from? The brain of the suckling rat is utilizing AcAc for cholesterol synthesis, and in the same myelinating cells that are making the VLCFAs through chain elongation would be generating cytosolic Ac-CoA from AcAc. That Ac-CoA generated from AcAc is probably then used to elongate FAs to make the VLCFAs needed for the sphingolipids of myelin and brain. What is likely happening during the suckling period is FAs in milk are being utilized as a substrate for making KBs and then the KBs are being used as the substrate for denovo lipid synthesis both cholesterol and FAs and for the elongation of FAs to make the VLCFAs. The carbon atoms involved in the elongation of the VLCFAs would preferentially come from the KBs. This is a metabolic process of lipid interconversion. One set of lipids are being converted into another set of lipids via oxidation to KBs, and then KBs are utilized for denovo lipid synthesis to produce a different set of lipids, the VLCFAs, and in the middle of the process is AACS. The carbon from milk fatty acids is being utilized for the synthesis of cholesterol and other isoprenoids, for the elongation of VLCFAs as well as for FA synthesis.

Another situation where lipid interconversion is likely to occur is with an adult on a vegetarian diet. These diets usually have an abundance of vegetable oils but totally lack cholesterol. The oils would be a good source for the generation of KBs. The liver would need to be making cholesterol for the creation of lipoproteins used to carry the TGs derived from the vegetable oils and other tissues such as skin and intestines would likely be synthesizing cholesterol as well. In liver and other tissues, the KBs are probably serving as a major substrate for the denovo synthesis of cholesterol via AACS. The data on the regulation of AACS with olive oil feeding and oleate perfusion of livers would support this role (32, 33). In this case of lipid interconversion, FAs of vegetable oils are being converted into cholesterol via the KBs and AACS probably in liver as well as in other tissues. Any high-fat diet may induce the utilization of KBs for cholesterol synthesis in many tissues, not just liver, as the data of Yamasaki *et al.* (39) on high-fat diet-induced obesity inducing KB utilization in bone osteoclasts suggest.

Denovo lipid synthesis from the KBs is likely to occur after meals containing medium-chain TGs such as are found in coconut oil (>50% MCFAs) and palm kernel oil (>50% MCFAs) and dairy products like butter, milk, yogurt, and cheese. The MCFAs are poor substrates for FA elongation, and so their major fate is fatty acid oxidation and the generation of KBs. Medium-chain TGs have been shown to stimulate hepatic Ac-CoA carboxylase, and increase in the synthesis of hepatic FAs is measured with ^3^H_2_O (96). Newly formed KBs derived from MCFAs then could be utilized for the denovo synthesis of both cholesterol and FAs via the AACS pathway in liver and other tissues. Another likely case of lipid interconversion is via the KBs.

Another interesting case of likely lipid interconversion via the KBs that was discussed above involves pregnancy and the growing fetus. In this situation in rats, it appears that FAs from the mother’s diet or from her TG stores are being converted to KB in her liver, then crossing the placenta into the fetus, and being incorporated into the lipids of the fetus (69). This is lipid interconversion with maternal FAs being converted to fetal lipids via the KBs.

The data suggest that lipid interconversion via the KBs and AACS is a process that occurs and clearly plays a role in the infant while suckling and can be important in cholesterol synthesis in the adult liver and other tissues. Diet, particularly the lipid and carbohydrate content, probably has an impact on how important and where and when this process takes place. Because the process produces cytosolic AcAc-CoA first, it may impact isoprenoid (not just cholesterol) synthesis in some tissues. The importance of this process in some lipogenic tissues such as skin, intestinal mucosa, mammillary gland, and adipose tissue is an open question and should be investigated. The quantitative contribution of lipid interconversion in various situations is not clear and is an area for further research.

A consequence of utilizing KBs for lipid synthesis is that when the KBs are used, glucose utilization is spared. Any situation where glucose is in short supply and there is a need for lipid synthesis, such as with a low carbohydrate diet, the KBs may be filling that need. Diet and the nutritional state of the animal may have a major influence on the utilization of KB for lipid synthesis in liver and other tissues. The role of KBs for lipid synthesis is probably reduced in the rat or mouse fed a traditional chow diet that is carbohydrate rich. It will also be reduced in cells and perfused organs that are grown in or perfused with high glucose media or high glucose perfusates and in the absence or near absence of KBs. The suckling rat is consuming a diet rich in fat and protein and low in carbohydrate, and the KBs are major substrates for lipid synthesis in numerous tissues of the suckling animal (91). The suckling rat also is using KBs as energy substrates in many tissues; both utilizations, anabolic and catabolic, of the KBs will be glucose sparing. Perhaps, the adult animal or humans on a high protein, high fat, low carbohydrate diet will also be using the KBs as major substrates for lipid synthesis.

## ACLY inhibitors

When the KBs are utilized for lipid synthesis via AACS, ACYL is being bypassed. An inhibitor of ACLY, bempedoic acid, has been developed and is approved as an adjunctive cholesterol-lowering agent (24, 25, 97). In addition, ACLY is being pursued as a target for the development of anticancer agents (23, 26) and other diseases of lipid metabolism such as nonalcoholic fatty liver disease (22). The question that needs to be asked, if the metabolic situation, the tissue, or dietary regime is such that KB utilization for denovo lipid synthesis via AACS is occurring and ACYL is being bypassed (partially or fully), will these ACLY inhibitors still work? Inhibiting ACYL with bempedoic acid, an approved adjunctive cholesterol-lowering agent (97), may only partially block the production of Ac-CoA and AcAc-CoA, the starting substrates for denovo lipid synthesis. Since it is potentially supplying just a partial block of cholesterol synthesis, it would probably be less effective in lowering cholesterol than an inhibitor such as the statins that block the critical enzyme in the cholesterol synthesis pathway. The active utilization of KBs for denovo lipid synthesis in permanent cell lines and Morris hepatoma 7777 cells suggests that some cancer cells have a significant capacity to utilize the KBs for denovo lipid synthesis (15). This could bypass ACYL in these cells and provide for the synthesis of the lipids needed for the cancer cells to grow and divide. In such cells and tumors, will an ACYL inhibitor, which may only be providing a partial block of denovo lipid synthesis, still work as an anticancer agent?

A consideration with bempedoic acid and possibly related compounds is that bempedoic acid is an inactive prodrug (24, 25). It first must be coupled to CoA to make bempedoyl-CoA to inhibit ACLY. The enzyme attaching CoA to bempedoic acid is almost exclusively located in liver, so inhibition of hepatic lipid synthesis is targeted (24). As was discussed earlier, palmitoyl-CoA and other acyl-CoAs, which are not that dissimilar from bempedoyl-CoA, are potent inhibitors of AACS (38). This raises the question of whether bempedoyl-CoA could be an inhibitor of AACS as well as an inhibitor of ACLY? Both reactions (AACS and ACLY) are similar, both use ATP, CoASH, and a carboxylic acid as substrates, both produce an acyl-CoA. If bempedoyl-CoA does inhibit AACS, bempedoic acid would be an inhibitor of both the AcAc and the citrate pathways for denovo lipid synthesis. The effects of bempedoyl-CoA on AACS should be investigated.

## Conclusions

The following conclusions were drawn from this review of AACS and the utilization of KBs for lipid synthesis.

1. An anabolic role for the KBs is their utilization for denovo lipid synthesis. It is a process that goes through a high-affinity enzyme, AACS, that does not require a high AcAc concentration, and this process is probably more important in the fed state than in starvation or diabetes.
2. Because AACS is found in most (if not all) lipogenic tissues, denovo lipid synthesis can start with AcAc and AACS as well as starting with citrate and ACLY in most if not all lipogenic tissues and cells.
3. AACS is a highly regulated enzyme in lipogenic tissues. It can produce AcAc-CoA and Ac-CoA for denovo synthesis of lipids. In many situations, it appears to be regulated in conjunction with the need for cholesterol, but in adipose tissue and maybe other situations, its regulation seems more tied to fatty acid synthesis. It has been shown to be regulated by gene expression, allosteric regulation, and there is some evidence that there may be active/less active forms of the enzyme. Some aspects of its regulation probably have not been discovered, regulation by diet, by hormones, and one aspect, in particular, is its regulation in conjunction or opposition to the regulation of ACLY.
4. The KBs are readily utilized as substrates for lipid synthesis in numerous lipogenic tissues, including liver, adipose tissue, lactating mammary gland, skin, intestinal mucosa, and adrenals. In numerous studied cases, they serve several-fold better than glucose as a substrate for denovo lipid synthesis, and when present, they often suppress the utilization of glucose for lipid synthesis.
5. KB utilization for lipid synthesis is particularly important in the suckling animal in brain, spinal cord, skin, and lung.
6. AcAc and AACS have a special role in cholesterol synthesis. This is a high-affinity pathway that allows AcAc to be directly incorporated into cholesterol. This may have an important role in cholesterol synthesis in liver and in a number of nonhepatic tissues that synthesize much of their own cholesterol, such as skin, intestinal mucosa, developing brain, and others.
7. Some cancer cells may have a high capacity (as compared with glucose) to utilize KBs for lipid synthesis.
8. Here, it is hypothesized that a major physiological role for the utilization of KBs for lipid synthesis is a metabolic process of lipid interconversion. Fatty acids are converted to KB in liver and kidney, and then the KBs are utilized to synthesize cholesterol, other fatty acids, the elongation of fatty acids, and other isoprenoids. The interconversion of fatty acids to cholesterol via the KBs and AACS may be a particularly important case of this lipid interconversion. This metabolic process would be important when the lipids in the diet or supplied to an organ do not meet the needs for specific lipids in that tissue.
9. Utilizing KBs for lipid synthesis is glucose sparing. It probably is more important in any situation where glucose needs to be spared such as in a low-carbohydrate diet and fats are plentiful.
10. What lipids and how much lipids in the diet should impact the metabolic process of lipid interconversion mediated by the KBs and AACS.
11. There probably is much left to be discovered about this metabolic process, how it is regulated, when it is important, what tissues are it important in, what lipids are it important for.

## Conflict of interest

The author declares no conflicts of interest with the contents of this article.
